# The Spatial Structure of Stimuli Shapes the Timescale of Correlations in Population Spiking Activity

**DOI:** 10.1371/journal.pcbi.1002667

**Published:** 2012-09-13

**Authors:** Ashok Litwin-Kumar, Maurice J. Chacron, Brent Doiron

**Affiliations:** 1 Program for Neural Computation, Carnegie Mellon University and University of Pittsburgh, Pittsburgh, Pennsylvania, United States of America; 2 Center for the Neural Basis of Cognition, Pittsburgh, Pennsylvania, United States of America; 3 Department of Physiology, McGill University, Montréal, Québec, Canada; 4 Center for Applied Mathematics in Biology and Medicine, McGill University, Montréal, Québec, Canada; 5 Department of Mathematics, University of Pittsburgh, Pittsburgh, Pennsylvania, United States of America; Research Center Jülich, Germany

## Abstract

Throughout the central nervous system, the timescale over which pairs of neural spike trains are correlated is shaped by stimulus structure and behavioral context. Such shaping is thought to underlie important changes in the neural code, but the neural circuitry responsible is largely unknown. In this study, we investigate a stimulus-induced shaping of pairwise spike train correlations in the electrosensory system of weakly electric fish. Simultaneous single unit recordings of principal electrosensory cells show that an increase in the spatial extent of stimuli increases correlations at short (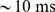) timescales while simultaneously reducing correlations at long (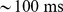) timescales. A spiking network model of the first two stages of electrosensory processing replicates this correlation shaping, under the assumptions that spatially broad stimuli both saturate feedforward afferent input and recruit an open-loop inhibitory feedback pathway. Our model predictions are experimentally verified using both the natural heterogeneity of the electrosensory system and pharmacological blockade of descending feedback projections. For weak stimuli, linear response analysis of the spiking network shows that the reduction of long timescale correlation for spatially broad stimuli is similar to correlation cancellation mechanisms previously suggested to be operative in mammalian cortex. The mechanism for correlation shaping supports population-level filtering of irrelevant distractor stimuli, thereby enhancing the population response to relevant prey and conspecific communication inputs.

## Author Summary

The size of a stimulus that is sensed by the nervous system can control the activity of neurons in sensory areas. How neural wiring supports this dependence remains an open question. We explore this general phenomenon using weakly electric fish, which possess a sensory system that detects electric field modulations produced by the surrounding environment. In particular, these animals' nervous systems are tuned to detect the difference between spatially compact prey inputs and spatially broad communication calls from other fish. In experiment, we discover that these two classes of stimuli differentially control the synchrony between pairs of electrosensory neurons. Using a computational model, we predict that this modulation is related to feedforward and feedback neural pathways in the electrosensory system, and we verify this prediction with experiments. This architecture prevents low frequency distractor stimuli, such as the animal's own tail motion, from driving neural population responses. With our model, we demonstrate how a common neural architecture enables a population-level code for behaviorally relevant stimuli.

## Introduction

There is a clear link between the combined activity of neurons and specific neural computations [1], [2]. A common observation from population recordings is that the correlation between the activities of pairs of neurons can be modulated – for instance, by the spatiotemporal structure of stimuli [3],[4], the perceptual state of the subject [5], [6], or the spatial focus of attention [7]–[9]. Theoretical work has focused on the cellular and circuit mechanisms that both determine and modulate correlation [10]–[20]. However, the general applicability of these theories is unclear [21], and how neural populations modulate the correlation between their spiking activity remains an open question.

One complication is that spike train correlations reflect common activity that may be measured at different timescales, ranging from a few (synchrony) to hundreds of milliseconds (co-variation of firing rates). For example, pairs of neurons in visual cortex [22], [23], olfactory bulb [24], and attention responsive cortical areas [7]–[9] show increases in spike time synchrony which accompany simultaneous decreases of rate co-variation. To indicate the complex temporal aspects of this modulation, we label a differential change in correlation over distinct timescales *correlation shaping* [19], [24]. In this study, we use a combination of *in vivo* recordings and computational modeling of electrosensory neurons to study how the spatial structure of a stimulus shapes the correlation of primary sensory neurons.

Weakly electric fish detect perturbations of their self-generated electric field through an array of electroreceptor neurons scattered on their skin surface which synapse onto pyramidal neurons within the electrosensory lateral line lobe (ELL) [25]. Relevant stimuli can be broadly categorized as either *local*, stimulating only a small fraction of the skin, or *global*, projecting to a broad area of the animal's body. Local inputs are a reasonable approximation to the spatial scale of prey inputs, while global inputs mimic communication calls from conspecifics [26]. We recorded simultaneously from pairs of ELL pyramidal neurons and found that global inputs increased spike train correlations at short timescales while simultaneously decreasing correlations at long timescales, when compared to the spike train correlation induced by local inputs. While there is a general understanding about how local and global stimuli control single neuron responses [26]–[30], the cellular and circuit mechanisms that allow the spatial extent of stimuli to shape correlated population activity in the electrosensory system are a new area of study.

Based on the well-characterized anatomy and physiology of electrosensory circuits [25], we developed a spiking network model of ELL pyramidal neurons that captured the experimentally observed correlation shaping. Diffuse inhibitory feedback was activated preferentially by global stimuli and provided a decorrelating signal that reduced correlations at long timescales. Further, global stimuli recruited feedforward circuitry that increased correlations at short timescales which were immune to feedback decorrelation. For sufficiently weak stimuli, we use a linear response framework [28], [31] to show how correlation shaping is consistent with a shaping of the single neuron stimulus-response gain function. We tested our model predictions experimentally by selectively blocking feedback input, causing spike train correlations at long timescales to increase, rather than decrease. This directly demonstrates how inhibition can be a source of decorrelation to pyramidal neurons, rather than a source of synchrony as described in many previous studies [10], [11], [32]–[35]. Finally, we used our understanding of the population's response properties to study how feedback selectively attenuates responses to distractor stimuli, improving the system's ability to represent relevant signals. In total, our results reveal novel principles by which feedforward and feedback neural circuits are differentially activated by stimuli to shape population spike train correlations.

## Methods

### Ethics Statement

Animals were obtained from local importers and were acclimated to the laboratory as per published guidelines [36]. All experimental procedures were approved by the McGill University Animal Care Committee and have been described in detail elsewhere [37].

### Electrophysiology

Briefly, dual extracellular recordings from the lateral and centrolateral ELL segments of *Apteronotus leptorhynchus* were made using metal-filled micropipettes [37]. Pyramidal cells within these segments can be distinguished from cells within the centromedial segment based on recording depth, the medio-lateral and rostro-caudal positions of the recording electrode with respect to surface landmarks such as the “T0” vein and its afferent veins [38], and their responses to sensory input as previously described [39]. Superficial pyramidal cells were identified based on their low (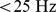) whereas deep cells were identified based on their high (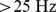) mean firing rates in the absence of EOD modulations [26],[30],[40]. All data was sampled at 10 kHz.

Random amplitude modulations of the animal's electric organ discharge (EOD) consisting of white noise low-pass filtered with a cutoff of 120 Hz (8th order Butterworth filter) were presented either globally via two electrodes positioned on either side of the animal or through a dipole located close to the skin surface [37]. The stimulus lasted 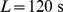 and consisted of 6 concatenated segments of the same frozen noise epoch that lasted 20 s [37].

Pharmacological blockade of the indirect feedback from EGp was performed by either applying the non-NMDA glutamate receptor antagonist CNQX within the ELL molecular layer [30] or by applying a 2% lidocaine solution to the praeminential-cerebellar tract (PECB) as done previously [41]. Since both manipulations gave rise to similar effects, the data was pooled.

### Data Analysis

#### Spike train cross-covariance functions

The recorded signals from a pair of neurons in response to the stimulus 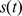 were thresholded in order to obtain the spike times 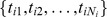, where 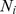 is the number of spikes from neuron 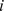 (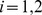). The spike train from neuron 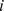 is then given by:
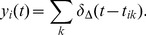
(1)Here 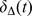 is the discrete approximation of the Dirac delta function with 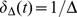 if 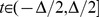 and is zero otherwise; throughout 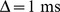 so that at most one spike was contained in any time window. We note that this is equivalent to discretizing time in bins of width 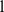 ms and setting the content of bin 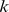 to 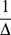 when there is a spike time 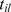 such that 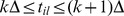 and to 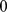 otherwise, as was done previously [30].

The firing rate for neuron 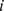 is then estimated as:
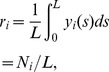
(2)where 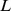 is the duration of a recording (typically 120 s). The spike train covariance at time lag 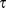 between neurons 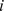 and 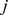 is defined as:
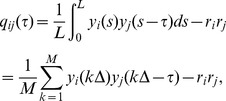
(3)where the number of time bins in the discrete spike train is 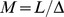. We refer to 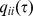 as the auto-covariance, while for 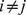 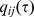 is called the cross-covariance.

#### Spike count correlations

We also considered the correlations between the spike counts of pairs of neurons. The spike count from neuron 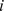 is simply defined as the number of spikes occurring in the time window 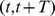. It is a random integer given by:
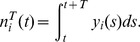
(4)For a given window size 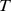, we computed a sequence of spike counts from neuron 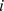 as 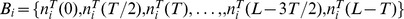, using overlapping windows to increase the number of estimates. We have that 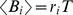, where 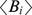 denotes the mean value of the sequence 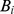. We can also obtain second order statistics from 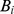 including the spike count variance and co-variance, which are defined by:
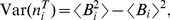
(5)

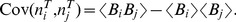
(6)


From these one can define the correlation coefficient between the spike counts 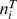 and 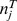 over a time window 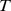:
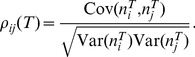
(7)We use 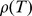 to denote the average value of 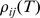 across all pairs 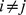 and similarly for other pairwise statistics. For small 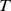, the correlation coefficient 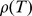 measures the degree of synchrony between the two trains, while, for large 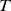, 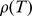 measures the co-variation in the firing rates of a pair of neurons [12], [13].

The variance and covariance functions of the spike count and spike train are related by:
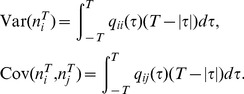
(8)These equations are the well known relations between second order spike count and spike train statistics [42], with 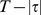 resulting from the convolution of the windowing function that converts spike trains to spike counts.

#### Within-trial vs. across-trial covariance functions and correlation coefficients

We note that both the spike train covariance function 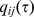 and correlation coefficient 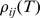 are within-trial measures of co-variability, since they incorporate both signal induced as well as trial-to-trial variable (i.e noise) aspects of common input fluctuations. Since we presented the same (i.e frozen) realization of the signal six times in succession, we were able to compute the spike train covariance and spike count correlation that were due purely to the common signal by computing joint statistics from neuron pairs recorded in different trials (i.e. across-trial). Specifically, denote the spike train of neuron 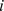 in response to the 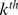 realization of the stimulus (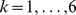) by 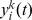. The across-trial spike train covariance between neurons 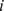 and 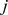 is then given by:
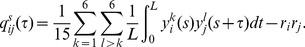
(9)In Eq. (9), 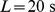. Eq. (9) measures the joint spike statistics from neuron pairs when the spike trains were not recorded simultaneously but were stimulated with the same signal. This is because the summation runs over all possibly non-repeating combinations (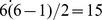) of the responses of each neuron to the six presentations of the frozen stimulus.

Similarly, one can define the spike count sequence for neuron 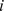 during stimulus realization 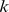 as 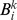. The across-trial spike count correlation coefficient between neurons 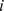 and 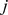 is then given by:
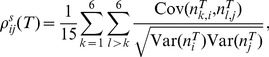
(10)where Cov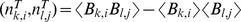 with 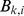 the sequence of spike counts from the 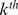 realization of the stimulus.

### Linear Response Approximation

We use linear response theory in order to derive an expression for the correlation coefficient 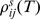 in terms of the stimulus gain, as done in past studies [12]–[14], [19], [28], [31], [43], [44]. We consider the Fourier transform of the spike train covariance function as the length of the trial 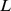 becomes large and assuming the processes are stationary:
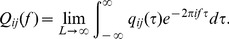
(11)Throughout, we will refer to 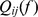 with 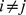 as the cross spectrum and 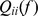 as the power spectrum. To relate spike count statistics to spike train statistics, we use the Wiener-Khinchin theorem to rewrite Eq. (8) (assuming 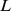 is large):
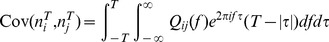
(12)

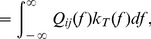
(13)with 
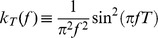
. Note that 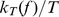 approaches a 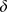-function centered at 0 as 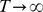 and a constant function on 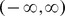 as 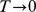. Therefore, for large 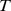, only the zero-frequency components of the spectra contribute to the integral, while for small 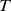, all frequencies contribute. A similar relation holds between 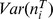 and 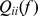.

For a fixed stimulus 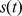, we assume that [13], [28], [31], [43]:
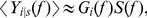
(14)where 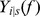 is the Fourier transform of the mean-subtracted spike train 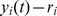 given a particular realization of 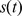, 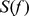 is the Fourier transform of the stimulus, and 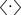 denotes an expectation over repeated presentations of the stimulus. 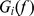 is the single neuron stimulus-response gain of the neuron (which we refer to as the *stimulus gain* for brevity). It relates the amplitude of the response to that of a signal at a particular frequency. For both experimental data and numerical simulations, we compute 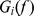 as:
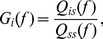
(15)where 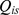 is the cross spectrum between 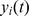 and 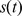 and 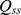 is the power spectrum of the signal.

Assuming that the spike trains are conditionally independent given the stimulus, we can write 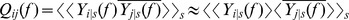, where 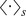 denotes an expectation over the random stimulus. Substituting Eq. (15) into Eq. (14),
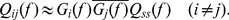
(16)Finally, combining Eqs. (13) and (16) yields the following approximation:
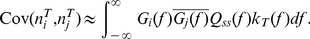
(17)
Eq. (17) relates the joint spike count variability to the stimulus gain 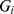, and has been derived in several past studies [13], [19]. We can then approximate the predicted across-trial correlation as:
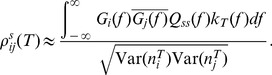
(18)


### Modeling

#### ELL anatomy

The neuroanatomy and physiology of the electrosensory system have been extensively characterized [25]. Pyramidal neurons in the ELL are subdivided according to several criteria. Roughly half of all pyramidal neurons have a basilar dendritic tree (BP neurons) and receive direct electrosensory afferent input. The other half lack a basal dendrite (nBP neurons) and receive afferent input only indirectly via interneurons [45]. Both BP and nBP neurons have an apical dendritic arbor; however, the extent of the arbor is variable across neurons. Pyramidal neurons with small apical dendritic trees are called deep neurons and do not receive much feedback input [30], [45], [46]. In contrast, pyramidal neurons with large apical dendritic trees are called superficial neurons and receive large amounts of feedback [30], [45], [46]. It has been recently shown [45] that the spatial projection of electroreceptor input to individual pyramidal neurons establishes a putative column, composed of BP and nBP deep and superficial pyramidal neurons.

The afferent and efferent projections between the ELL and higher brain structures further distinguish ELL pyramidal neurons. Indeed, only deep pyramidal neurons project to the praeminentialis dorsalis (Pd) [46], a second order isthmic structure that directly projects to the posterior eminentia granularis (EGp), which in turn projects back to the ELL along the dorsal molecular layer via parallel fibers [25] that make synaptic contact onto the large apical dendritic trees of superficial pyramidal neurons. Thus, the deep ELL
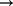
EGp
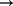
superficial ELL feedback pathway can be characterized as open-loop [46]. Electrophysiological studies suggests that EGp granule cells show temporal locking to electrosensory input [46], [47] and that the indirect feedback input onto ELL pyramidal neurons is in the form of a negative image of the stimulus that is activated by spatially diffuse but not by spatial localized stimuli [30], [46].

#### ELL model description

Our model of the deep pyramidal neuron to superficial ELL feedback via the nP and EGp contained three distinct neural populations: a deep (Dp) ELL population that projected to a population of granule cells in the EGp, which in turn provided feedback to a population of ELL superficial (Sf) neurons. All cells were modeled with leaky integrate-and-fire (LIF) dynamics [48]. Numerical values of model parameters can be found in Table 1, and a detailed model summary [49] can be found in Table S1. The membrane potential 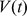 obeyed linear subthreshold dynamics supplemented with a spike-reset rule so that 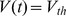 implied that 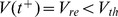, and 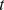 was marked as a spike time. The deep population consisted of 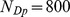 neurons, and the membrane potential of the 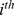 deep neuron obeyed:
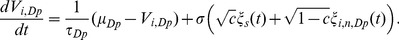
(19)The first two terms of the right hand side of Eq. (19) model a static rest state and an intrinsic leak process, respectively. The process 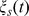 models Gaussian stimulus locked electroceptor activity, while 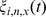 models stimulus independent activity afferent to neuron 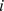 in population 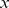 (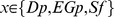). As in the experiments, we set 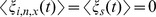, but the temporal structure of the processes was white with 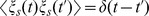, 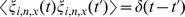, and 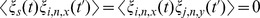 for 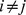 or 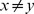. The electroreceptor input contrast was set by 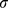 and the correlation of the stimulus locked component by 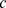.

**Table 1:** Parameter values used in numerical simulations.

Parameter	Description	Value
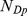	Number of deep neurons	800
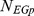	Number of EGp neurons	200
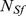	Number of superficial neurons	2
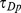	Deep membrane time constant	10 ms
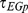	EGp membrane time constant	10 ms
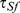	Superficial membrane time constant	15 ms
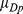	Deep bias	−56 mV
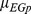	EGp bias	−60 mV
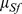	Superficial bias	−56 mV
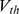	Threshold voltage	−55 mV
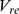	Reset voltage	−65 mV
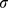	Noise strength	1 mV
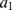	Deep to EGp synaptic strength	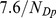 mV
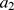	EGp to Superficial synaptic strength	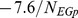 mV
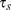	EGp to Superficial synaptic time constant	5 ms
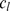	Local input correlation	0.1
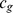	Global input correlation	0.2

The EGp population consisted of 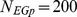 neurons, and the membrane potential of the 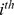 EGp granule cell followed:
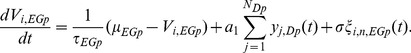
(20)Here 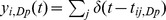 is the spike train from the 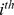 deep neuron, and 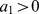 is the strength of excitation from the Deep ELL
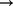
EGp. The time constant 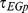 was chosen as 10 ms, based on recent measurements of input resistance for these cells of approximately 2 G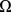 [47] and data from cerebellar granule cells indicating typical capacitance values of 3–5 pF [50]–[52].

Finally, since we are only interested in the pairwise correlation between superficial neurons and because the feedback is open-loop, it is only necessary to consider a pair of superficial pyramidal neurons. As such, we set 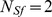. The 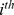 superficial pyramidal cell's membrane dynamics are given by:
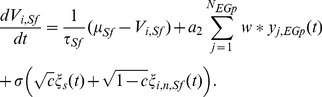
(21)Here 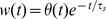 where 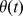 is the Heaviside function. The operation 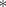 denotes convolution. The inhibitory coupling from EGp to the ELL was set by 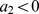.

During local stimulation, a fraction 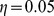 of deep neurons received coherent, stimulus-locked electroreceptor input (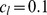), while all other deep neurons received uncorrelated input modeling spontaneous afferent activity. During global stimulation, all deep neurons (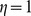) received stimulus-locked input (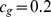). The increased value of 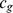 reflects the fact that global stimuli will spatially saturate the receptive field center and will thus more effectively drive the afferent population [29], [53].

In our model, a pair of neurons in a given layer could receive correlated input from the previous layer in two ways. First, a neuron in the previous layer could project to both downstream neurons and thus correlate their input. Second, neurons in the previous layer could become locked to the stimulus and their pooled activity could correlate the downstream neurons, even if their projections did not overlap anatomically. In the linear model, we assumed that the first source of common input is negligible relative to common input from stimulus locked, pooled activity, as is often the case in feedforward networks [54]. Consequently, correlations between model neurons were due only to external signals that synchronously recruited electroreceptors. Therefore, 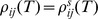 for the model.

To evaluate 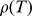 for our model using the linear response approximation, we computed the superficial neuron stimulus gain 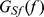. For numerical simulations, we estimated 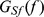 using Eq. (15). However, following past work [28], [31], we derived a theoretical approach to compute 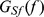. For global stimulation and assuming that both the input correlations 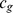 and the effective coupling 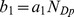 and 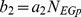 are sufficiently small, we compute the feedback filter from the Deep ELL
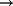
EGp
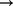
Superficial ELL using the serial computation
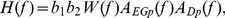
(22)where 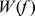 is the Fourier transform of the exponential synaptic kernel 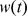. This result follows simply from the linear convolution of Deep ELL activity to EGp and then from EGp activity to superficial ELL through 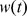. Here we have introduced 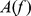, the single neuron cellular response function (which we refer to as the *cellular response* for brevity) that measures a neuron's response to an applied current, independent of network feedback. 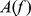 can be computed using standard techniques from statistical mechanics (see Text S1).

We note that 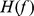 can be calculated for mixed excitatory and inhibitory feedback to superficial neurons. In this case, the value of 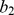 should be interpreted as the effective input strength from both excitatory and inhibitory populations. For example, if the fraction of excitatory synapses from EGp to superficial neurons is given by 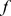 and the synaptic strength of excitation and inhibition are 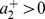 and 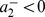, respectively, then we have 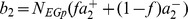. Previous studies have established that the stimulus-locked EGp feedback is net inhibitory [46], and we therefore model the pathway as purely inhibitory for simplicity.

With 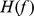, we calculate the stimulus gain of a superficial ELL neuron 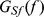 as given in Eq. (25). Further, these techniques also permit a calculation for the power spectrum 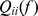. With theoretical expressions for 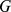 and 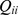, and assuming the signal is Gaussian white noise with unit variance, we use Eqs. (17) and (18) to obtain a theoretical prediction for the spike count correlation between the two superficial ELL neuron spike trains:
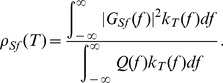
(23)Here we have used the homogeneity of the spike trains to set 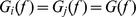 and 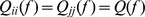 for all superficial neurons.

## Results

### Correlation Shaping with Global and Local Stimuli

**Figure 1:**
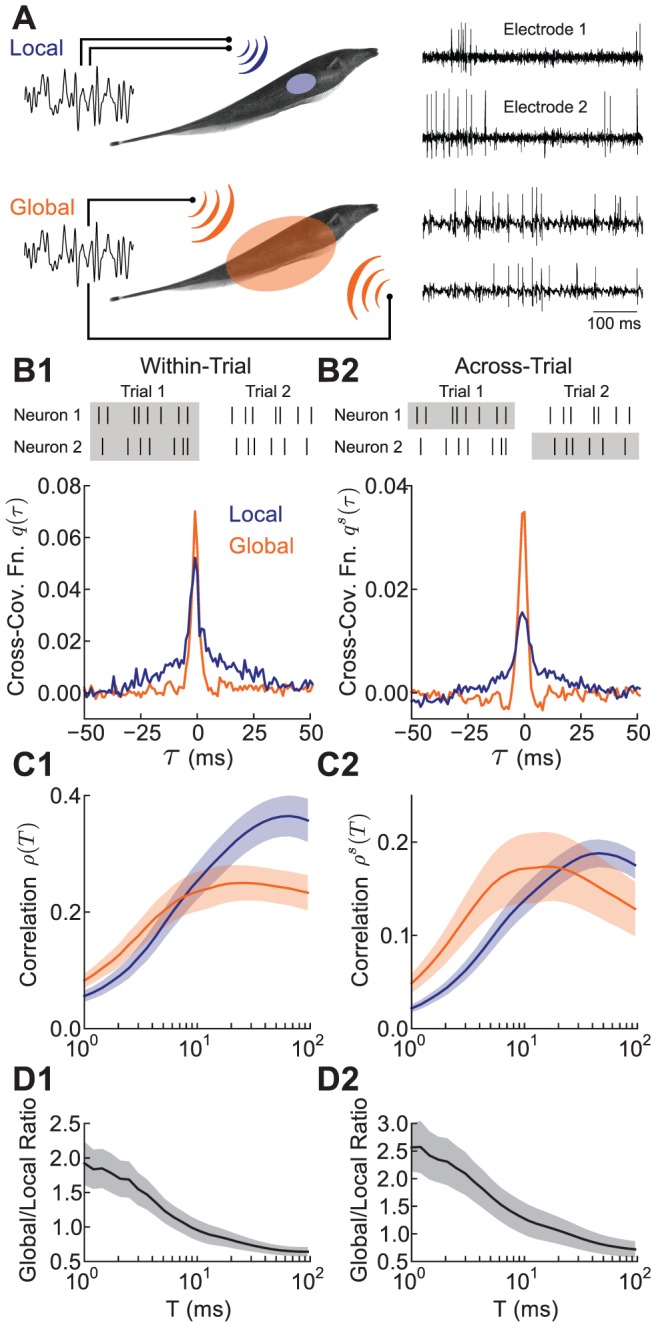
The spatial extent of electrosensory stimuli shapes the temporal correlation between the spike times from pairs of ELL pyramidal neurons. **A**, Stimulus protocol for local and global stimulation. Left: Gaussian distributed electric field stimuli with broadband spectral content (uniform over 0–120 Hz) were applied in a spatially compact (local) or diffuse (global) manner. Right: Paired extracellular recordings of ELL pyramidal neurons were made during stimulation. **B1**, Spike train cross-covariance function in the local and global stimulus configuration for pairs of simultaneously recorded superficial BP neurons (within-trial correlation). Correlation function is normalized by firing rate. **B2**, Same as B1 except computed between spike trains recorded during distinct trials. **C1**, Within-trial spike count correlation as a function of window length (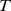) in the local and global stimulus configuration. **C2**, Across-trial spike count correlation as a function of window length 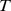 in the local and global stimulus configuration. **D1**, Ratio of global and local within-trial spike count correlations shown in panel C1. **D2**, Ratio of across-trial global and local spike count correlations shown in panel C2. The data set consists of n = 10 pairs of neurons, and all curves are population average quantities. In all panels, shaded regions denote 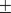 standard error.

We examined the response of ELL pyramidal neurons to time-varying electrosensory input. Broadband electrosensory stimuli (Gaussian, 0–120 Hz) were applied to awake, behaving weakly electric fish (*Apteronotus leptorhynchus*; see Methods). Throughout the study, we delivered stimuli in one of two spatial arrangements: a *local* or *global* configuration [26], [27], [29]. In the *local* configuration, stimuli were spatially compact, delivered through a small dipole (tip spacing of 2 mm), and excited only a small region of the skin surface (Figure 1A, left, blue). Local inputs mimic prey stimuli which drive only a spatially localized portion of the receptive field of an ELL pyramidal neuron [55]. In the *global* configuration, stimuli were spatially broad, delivered through a pair of electrodes located on each side of the animal, and affected the entire surface of the animal (Figure 1A, left, orange). Global inputs mimic stimuli caused by conspecifics which drive nearly the entire surface of one side of the animal, stimulating both the classical and non-classical receptive field of a target pyramidal neuron [29], [56]. During both local and global stimulation, simultaneous extracellular recordings of ELL pyramidal neuron pairs were collected (Figure 1A, right). There was an intentional selection bias for superficial basilar pyramidal (BP) neurons [25], since these neurons are known to receive feedback projections that shape their responses to sensory input [30], [45], [46]. Superficial neuron firing rates in the local and global configurations were similar (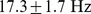 and 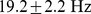 respectively).

We used the simultaneous unit recordings to estimate the spike train cross-covariance function (see Methods Eq. 3) for neuron pairs in both the local and global stimulus configurations. Global stimulation set a narrow peak of the cross-covariance function with a high maximum at zero lag, while it was broad with a lower peak value for local stimulation (Figure 1B1), consistent with previous reports [37].

To quantify this shift in covariance at different timescales, we computed the correlation coeffcient between the spike counts of neuron pairs' outputs [22], [42]. This provided a normalized measure of the similarity between the two spike trains as observed over windows over a particular length 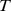 (see Methods Eq. 7). At small window sizes (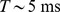), spike count correlation was larger during global stimulation than during local. For large window sizes (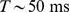), this relationship was reversed (Figure 1C1). Correlation 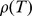 is generally a rising function of window size [57], since for small 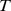 few spikes will occur in the same window. However, even small values of correlation (e.g. 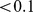 in magnitude) have substantial influence on the propagation of neural information [54], [58] and neural coding [59]. To provide a relative measure of the shift in correlation between the two states, we considered the ratio of global correlation to local correlation. This was a decreasing function of window size which was substantially greater than 1 for small window sizes and lower than 1 for large window sizes (Figure 1D1).

We performed statistical tests to confirm that the trends observed were significant. Nonparametric tests confirmed that the distributions for the local and global conditions were different (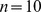, evaluated at 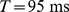, 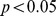, two-sample Kolmogorov-Smirnov test). The trends with timescale were also significant (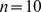, 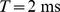 compared with 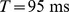, 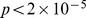 for local and 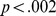 for global stimulation, two-sample Kolmogorov-Smirnov tests). The means of the distributions were also different (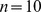, evaluated at 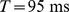, 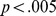, paired t-test). In summary, the spatial extent of the electrosensory signal shaped the timescales over which spike train pairs were correlated.

### Shifts in Single-Neuron Response Gain Predict Correlation Shaping

In general, correlated neural activity can be decomposed into stimulus induced and non-stimulus induced components [21], [60]. Stimulus induced correlations reflect the two neurons locking to a dynamic stimulus, while the non-stimulus induced correlations reflect the neurons sharing a portion of their trial-variable noise, presumably from a common pre-synaptic source. To uncover the cellular and circuit mechanisms underlying correlation shaping, we first determined whether the changes in correlation observed were present across trials and therefore related to how neurons responded to the repeated stimulus. Using spike trains across different trials of identical stimulus presentations, we computed the across-trial spike train cross-covariance functions and spike count correlations (Figure 1B2,C2; see Methods Eqs. 9, 10). The magnitude of these across-trial correlations was less than that of the within-trial correlations, indicating the presence of some trial-variable noise (compare Figure 1C1 and 1C2). Nevertheless, the differential shaping of correlations at short and long timescales was still present in the across-trial spike count correlation (Figure 1C2,D2). This suggests that the way stimulus processing shifts between local and global conditions is related to the mechanisms responsible for correlation shaping.

**Figure 2:**
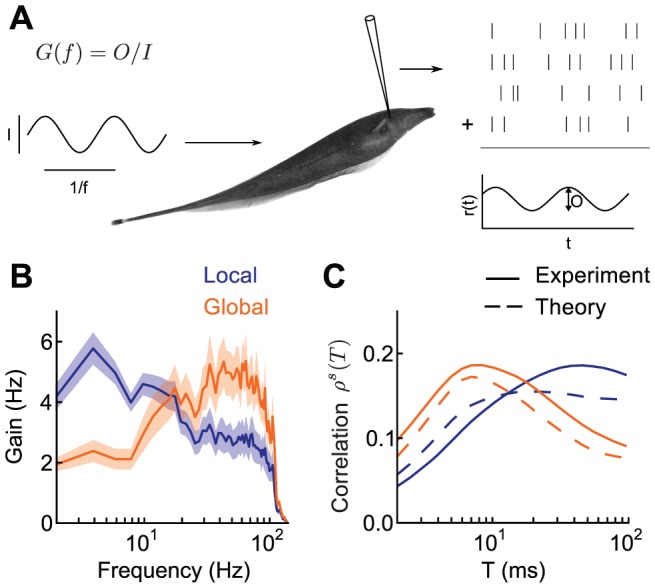
Shifts in stimulus gain predict spike train correlation shaping. **A**, Schematic illustration of stimulus gain. The gain 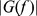 is described as the ratio of the change in the output firing rate 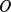 of a neuron that is evoked by an input sine wave stimulus of amplitude 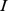. **B**, Gain for neuron pairs during local and global stimulation. The signal was assumed to have unit amplitude. **C**, Across-trial spike count covariance (solid) and the prediction from a linear response theory (dashed, see Methods Eq. 17), in both global and local stimulus conditions. The data set consists of n = 10 pairs of neurons, and all curves are population averages. In all panels, shaded regions denote 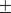 standard error.

To investigate this relationship, we considered the *stimulus gain* 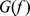, which measures a neuron's response to an external electrosensory stimulus at frequency 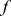 (Figure 2A, see Methods Eq. 15). We computed the gain in the two stimulus conditions and found that during local stimulation, the gain function was low-pass, while during global stimulation, it was high-pass (Figure 2B), consistent with previous studies [29], [30]. We then asked if the observed changes in correlation could be related to this shift in frequency selectivity.

Motivated by past studies [12], [13] we assumed that the cross-spectrum between the spike trains was proportional to the product of their stimulus gain functions (see Methods Eq. 16). This amounts to assuming that the common stimulus is the only source of correlation in the neural responses. This theory predicts that the correlation for large window sizes 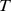 is determined by stimulus gain at low frequencies. In contrast, correlation for small windows involves gain at high frequencies. The shift in 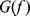 from low frequency transfer for local inputs to high frequency transfer for global inputs therefore implies global stimulus correlation will be enhanced for small 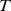 and attenuated for large 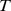, with the inverse true for local stimulation. We verified this hypothesis, obtaining a prediction of the spike count correlation in the two states that matched the experimental data (see Methods Eq. 18; Figure 2C, solid versus dashed curves). Thus, the shift in the frequency-selectivity of superficial neurons' stimulus gain between the local and global conditions indeed predicted the changes in correlation.

### Modeling ELL Pyramidal Cell Responses

**Figure 3:**
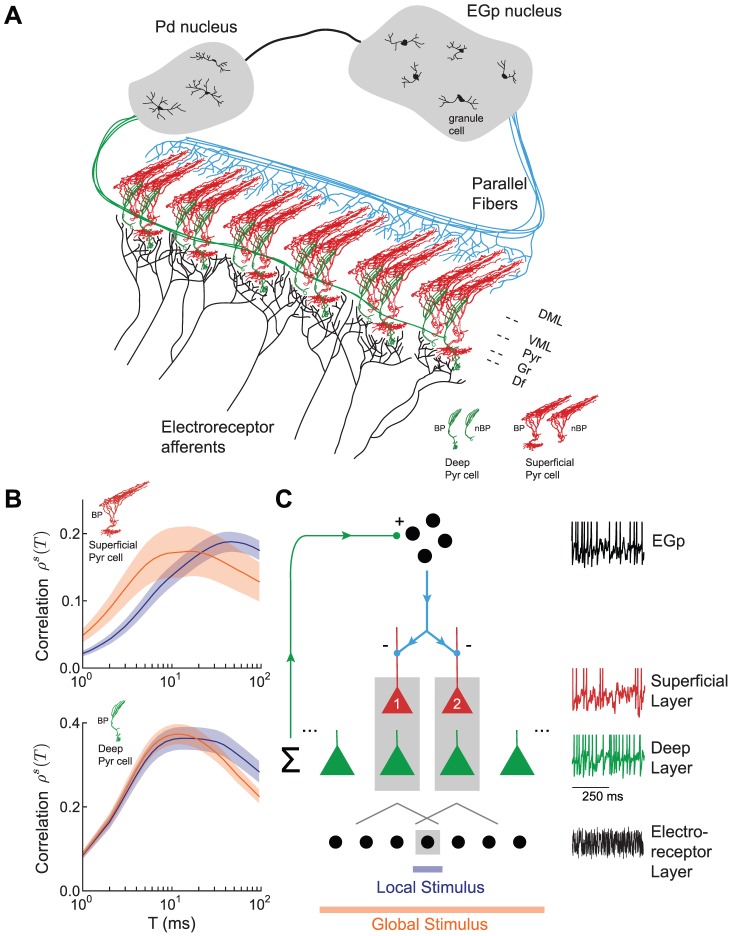
Open loop feedback inhibition in electrosensory neural circuitry. **A**, Detailed schematic of peripheral neural circuitry in the electrosensory system. Basilar (BP) and non-basilar (nBP) pyramidal neurons in the electrosensory lateral line lobe (ELL) have their somata located in the Pyramidal cell layer (PCL). Deep pyramidal neurons (green) have small apical dendritic arbors, projecting only to the Ventral Molecular Layer (VML). In contrast, superficial pyramidal neurons (red) have large apical dendritic arbors, projecting to the Dorsal Molecular Layer (DML). Pyramidal neurons receive direct and/or indirect input from feedforward electroreceptor afferent input to the Deep Fiber Layer (DFL). Deep pyramidal neurons excite neurons in the praminentialis dorsalis (Pd), which in turn excite granule cells in the posterior eminentia granularis (EGp). The EGp projects parallel fiber feedback along the DML exclusively targeting ELL superficial pyramidal neurons. In total the deep ELL
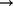
EGp
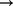
superficial ELL pathway is an open loop feedback structure. Pyramidal neuron graphics were from example neurolucida traced neurons [46]. **B**, Stimulus correlation for pairs of experimentally recorded deep pyramidal neurons (n = 45 pairs; 10 neurons were used) that were driven by the stimulus in local and global (bottom). Little correlation shaping is present. For comparison purposes we show the stimulus correlation for pairs of superficial neurons (top, Figure 1C2). **C**, Simplified model of the ELL-EGp circuit. Individual neurons in the deep ELL, EGp, and superficial ELL were modeled with leaky integrate-and-fire neuron dynamics (example realizations on right). Electroreceptor input was modeled as white noise, with 5% of deep pyramidal neurons receiving a stimulus-locked component in local and 100% in global. We studied the spike responses the pair of superficial pyramidal neurons (labeled 1 and 2) that receive both afferent and EGp feedback inputs.

To understand mechanisms behind the shift in neuronal responses under the local and global stimulus conditions, we constructed a simplified population model of ELL pyramidal neurons based on known anatomical and functional data as well as our experimental results (Figure 3A; for a detailed discussion of the anatomy, see Methods). This model captured two generic circuit features that modulated population responses: feedforward sensory input and feedback inhibition. All pyramidal neurons received feedforward electrosensory input via electroreceptors, but were divided into two classes based on their feedback afferents: deep neurons did not receive feedback from higher regions, but superficial neurons did receive inhibitory feedback. This feedback arrived from the posterior eminentia granularis (EGp), which was in turn innervated by the deep neurons. In total, this structure formed an open-loop inhibitory feedback pathway, from deep neurons to EGp neurons to superficial neurons. Motivated by past studies, ELL pyramidal neurons were modeled as simple leaky integrate-and-fire units [27], [28], [46]. Consistent with experimental data [30], superficial firing rates in the model were lower than deep firing rates (12 Hz and 36 Hz, respectively) in both local and global stimulation conditions.

Previous studies have shown that EGp feedback modulates both the static [41] and dynamic [30] gain of single neuron responses. However, how it controls the ELL population response, and in particular correlations between pyramidal neurons, is unknown. To determine whether feedback is responsible for stimulus-dependent correlations, we recorded from deep pyramidal neurons receiving a frozen stimulus and computed stimulus correlations between the pairs of spike trains. Consistent with the lack of feedback projections to this subpopulation, these neurons did not show substantial shaping of correlations between the local and global conditions (Figure 3B, bottom), in contrast with superficial pyramidal neurons (Figure 3B, top). The small decrease in correlation for large time windows observed during global stimulation for deep neurons (Figure 3B, bottom) is consistent with these neurons receiving little feedback input [40].

### Recruitment of Feedback in the Model During Local and Global Stimulation

**Figure 4:**
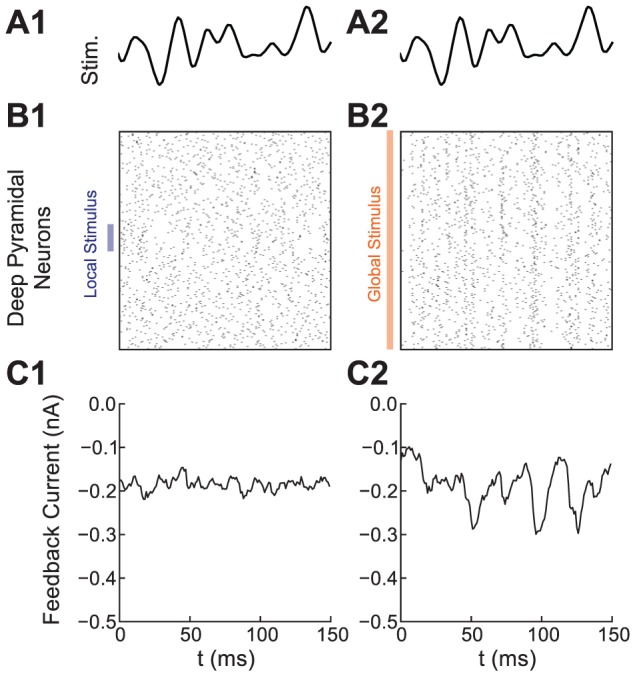
Model EGp feedback is stimulus locked for global, but not local, stimulation. **A** Low-pass (0–60 Hz) filtered version of the electrosensory stimulus. Filtering was done as a visual aid in relating the stimulus to the feedback in (C2). **B1**, Raster plot of the deep neuron population during local stimulation. The signal weakly correlated only a small fraction of the population. **B2**, Same as (b1), but during global stimulation. The spatially broad stimulus correlated the entire deep population. **C1**, EGp feedback current during local stimulation, showing little stimulus locking. **C2**, EGp feedback was stimulus-modulated by the global signal, due to recruitment of the deep population by the stimulus. The inhibitory feedback is a negative image of the stimulus (A2).

We used our model to examine the stimulus dependence of EGp feedback. In our model, electrosensory stimulation caused the firing of deep pyramidal neurons to become stimulus-locked. When the stimulus was local, only a small fraction of this population was stimulus-locked, so that the average correlation across the deep population was low (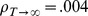 across the population, Figure 4B1). The weak stimulus correlation across the deep population failed to recruit coherent activity in the EGp granule cell population, resulting in a near tonic inhibitory feedback to the ELL (Figure 4C1). In contrast, when the stimulus was global, the entire deep population was correlated by the stimulus (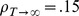, Figure 4B2). This led to a dynamic, stimulus locked EGp feedback to the superficial neuron pair (Figure 4C2). Thus, our model captured a link between the temporal locking of EGp feedback and the spatial extent of the external stimulus, which has been suggested in past experiments [46], [47].

Having characterized the EGp feedback, we next determined how it shaped the responses of superficial neuron pairs. The total input to a model superficial pyramidal neuron, from both feedforward and feedback sources, is:
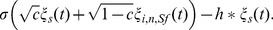
(24)Here 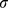 is the strength of the afferent activity to an ELL pyramidal neuron and 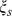 and 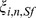 are Gaussian white noise processes modeling stimulus locked and unlocked (noise) afferent inputs, respectively. The parameter 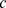 is the fraction of receptor afferents that are stimulus-locked, which determines the correlation between the electroreceptor input to neuron pairs. The function 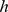 is the parallel fiber feedback kernel and involves compound processing of the stimulus by the population of deep ELL neurons, the EGp granule cells, and finally the inhibitory feedback pathway from the EGp to the ELL (see Methods Eq. 21). Assuming weak stimulus correlations (small 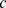) and weak EGp feedback, we use linear response theory [28], [31], to obtain an expression for the stimulus gain of a superficial pyramidal neuron (see Methods):
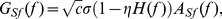
(25)Here 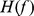 is the Fourier transform of the feedback kernel 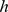 (see Eq. 22 in Methods), and 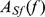 is the *cellular response* of a superficial neuron, which measures its response to a fluctuating current applied directly to the neuron (see Eq. 8 in Text S1). In contrast to the stimulus gain, the cellular response does not depend on network feedback. The parameter 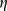 is the spatial extent of the stimulus (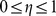), with 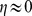 modeling the lack of stimulus-coherent EGp feedback for local stimulation, and 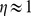 the full recruitment of EGp feedback for global stimulation (Figure 4). With this model of how 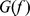 shifts between local and global stimulus configurations, we next build a theory for the correlation shaping within the superficial ELL pyramidal neuron population.

### Correlation Shaping in the ELL-EGp Network Model

**Figure 5:**
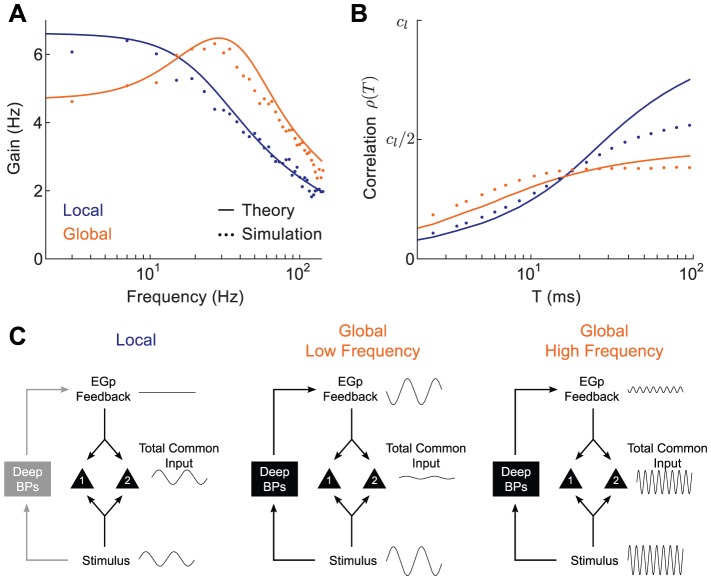
Model ELL-EGp network captures correlation shaping between local and global stimulation. **A**, Stimulus gain of superficial BP neurons in the model (compare to Figure 2B). Our analytical theory (solid) matches the simulation results from the ELL-EGp network (dots). **B**, Correlation between superficial BP neuron pairs during local and global stimulation of the model (compare to Figure 1C). Since our theory predicts a linear relationship between output correlation and input correlation, the output is shown in units of input correlation in the local state 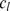, which was 0.1 in simulations. **C**, Idealized schematic illustrating the effect of feedback on shared fluctuations. Left: local inputs fail to recruit EGp feedback via deep population (see Figure 4), so common input arises purely through feedforward stimulus drive. Center: Low frequency global input recruits a negative image of the stimulus, which cancels the common input to the pair of superficial pyramidal neurons. Right: The cancellation signal is weak for high frequency global inputs due to the low-pass nature of the feedback. Hence, the common fluctuations are not cancelled.

We used our ELL-EGp network model to relate the spatial extent of an electrosensory stimulus and the timescale of the pairwise correlation between spike trains from superficial BP neurons. During local stimulation, pairs of nearby superficial neurons received correlated electroreceptor input (Figure 3C). The degree of correlation between the afferent input to the superficial pair was 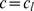. The EGp feedback did not exhibit a substantial stimulus-locked component (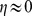) during local stimulation, and hence did not contribute to common fluctuations (Figure 4C1). Thus, the stimulus gain in the local condition, denoted 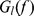, reduced to:
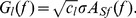
(26)Our theoretical 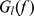 (see Methods) quantitatively matched estimates from simulations of the ELL-EGp network of leaky integrate-and-fire neurons (Figure 5a, blue curve and blue dots) and qualitatively matched the low-pass nature of 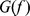 obtained from experiments (Figure 2B, blue). The calculation demonstrates that the gain to local stimuli of superficial pyramidal neurons is primarily determined by the cellular response 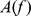, suggesting that feedback network dynamics can be ignored.

The lack of network activity for local stimulation (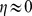), was contrasted with the recruitment of EGp feedback for global stimulation (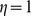). During global stimulation, we also assumed that the receptive fields of neurons were fully saturated, rather than being partially driven due to the limited extent of the stimulus, as suggested by experimental estimates [53]. We therefore increased the correlation of electroreceptor afferents in the global state, so that 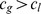. Combining these two model assumptions, we expressed the gain in the global configuration, 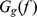, as:
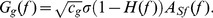
(27)If 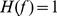 – that is, if the negative feedback were a perfect replica of the feedforward signal – the stimulus gain 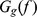 would be zero, indicating complete stimulus cancellation by the feedback pathway. However, since the negative feedback was low-pass due to neuronal integration and synaptic filtering along the feedback pathway, only the low frequency components of the gain were strongly attenuated. Consequently, 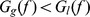 for sufficiently low frequencies (Figure 5A, compare orange and blue curves for 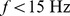). However, 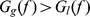 for high frequencies (Figure 5A, compare orange and blue curves for 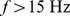), because of the increase in receptive field saturation (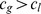). Our theoretical 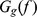 matched simulations of the ELL-EGp network (Figure 5A, orange curve and orange dots). Thus, the combination of feedback recruitment and feedforward saturation during global stimulation captured the experimentally determined shift in stimulus gain known to occur between local and global stimulation (Figure 2B and see [29], [30]).

Next, we examined how this gain shift controlled correlations across the population of superficial pyramidal neurons. Using the linear response theory we used to predict signal correlations in the experimental data (Figure 2, see Methods Eq. 23), we calculated theoretically the correlations between model pyramidal neurons. Global stimulation simultaneously increased short 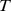 correlation and decreased long 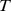 correlation compared to local stimulation (Figure 5B). These findings matched the experimental results (compare Figures 1C and 5B) and are the primary theoretical result of this study.

Our model provides clear intuition for how the combination of receptive field saturation and the recruitment of EGp feedback during global stimulation shapes the correlation of ELL pyramidal neuron activity (Figure 5C). During local stimulation, EGp feedback was not recruited and the feedback did not cancel the feedforward signal from the electroreceptors (Figure 5C, left). This case is contrasted with global stimulation, in which a broad stimulus-induced synchronization of all of the deep ELL neurons recruited a stimulus-locked EGp feedback. This feedback was low-pass, and therefore canceled the low frequency components of the signal (Figure 5C, middle), but not the high frequency components (Figure 5C, right). Thus, correlations due to global stimulation were canceled only for sufficiently long timescales 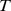 (Figure 5B, 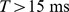). Furthermore, the saturation of the receptive field input (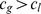) enhanced the correlation 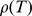 for small 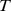 (Figure 5B, 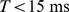). In total, feedforward and feedback circuitry shaped 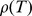 depending on the spatial profile of the electrosensory signal.

Our ELL-EGp network model distills correlation shaping into two hypotheses that link the *spatial* properties of an electrosensory stimulus and the *timescale* of pairwise correlation between the spike responses of ELL superficial pyramidal neurons:

1. Receptive field saturation for spatially broad signals increases the short timescale correlation between the spike trains from superficial pyramidal neurons.
2. Recruitment of EGp feedback by spatially broad signals decreases the long timescale correlation between the spike trains from superficial pyramidal neurons.

To study these two components of correlation shaping in isolation from one another, we used a combination of analysis on a subclass of ELL pyramidal neurons and pharmacological blockade of EGp feedback.

### Correlation Shaping of nBP Neuron Responses

We first tested how short timescale correlation was affected by receptive field saturation (Hypothesis 1). The ELL has two classes of pyramidal neuron: non-basilar pyramidal (nBP) and basilar pyramidal (BP) neurons, distinguished by the extent of their basilar dendritic arbor (Figure 3A). While BP neurons respond to positive deflections of the electric field, nBP neurons are oppositely tuned, due to their afferent inputs arriving solely via an inhibitory interneuron population [25]. This difference in the feedforward afferent architecture to nBP neurons compared to BP neurons produces nBP neuron classical receptive fields that are smaller than those of BP neurons [26]. Despite the difference in feedforward afferent input for BP and nBP neurons, both superficial BP and nBP neurons receive near equivalent feedback from EGp parallel fibers (Figure 3A). Thus, a comparison between BP and nBP neurons is sensitive to a difference in feedforward afferent drive, and not to EGp feedback. We hypothesized that global inputs would not drive nBP neurons as strongly as BP neurons because of their smaller classical receptive fields. Hence, short timescale correlation during global stimulation for nBP neurons should be less than for BP neurons.

**Figure 6:**
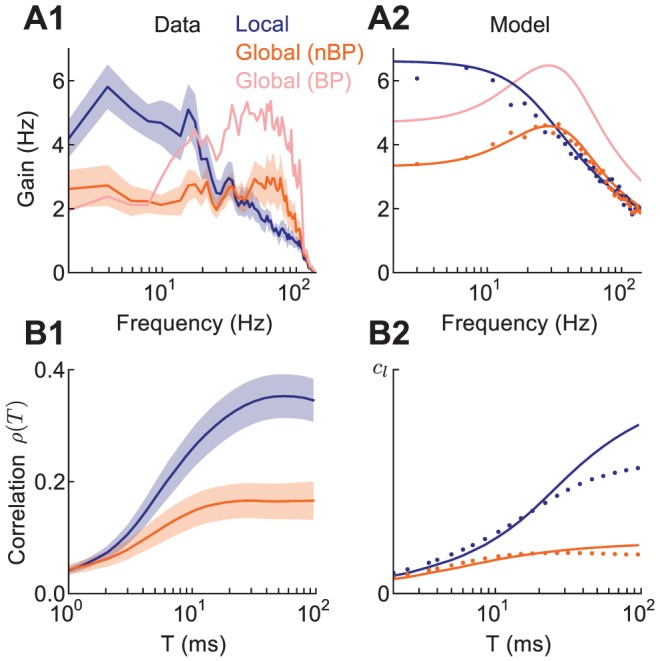
Saturation of the receptive field for both local and global stimuli makes short timescale response insensitive to the spatial extent of electrosensory stimuli. **A1**, Experimental stimulus gain for nBP neurons (n = 14) in local and global stimulus configurations. The gain for BP neurons in the global configuration is shown for comparison (see Figure 2B). **A2**, Stimulus gain for model nBP neurons (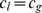) in local and global configurations, and the model BP neurons (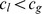) in global for comparison. **B1**, Recorded spike count correlation over windows of length 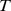 for pairs of nBP neurons. As with BP neuron pairs, firing rates in the local and global states were similar (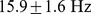 and 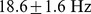, respectively). **B2**, Spike count correlation for pairs of model nBP superficial neurons in the ELL-EGp network. For the model results (A2,B2) our analytical theory (solid) matches the simulation results from the ELL-EGp network (dots). Values are shown in units of input correlation in the local state 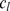.

We first calculated the stimulus gain for nBP neurons. The difference in gain between local and global stimuli for nBP neurons was different than that for BP neurons (Figure 6A1; [30]). In particular, while nBP and BP neurons both exhibited a reduction in low frequency gain during global stimulation, nBP neurons exhibited little enhancement of high frequency response. Our model network replicated this difference (Figure 6A2) when we assumed that the nBP neurons integrate stimuli over smaller regions of space, such that local inputs saturate the receptive field (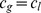), in contrast to the BP neuron case (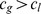). The lack of high frequency shaping of gain for nBP neurons across local and global configurations predicts that the small 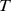 correlations do not substantially increase in the global state, while EGp feedback still attenuates low frequency gain and hence large 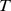 correlations. Measurements of 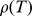 for nBP neurons in both the ELL-EGp model (Figure 6B2), as well as nBP neurons recorded *in vivo* (Figure 6B1) supported this prediction. Thus, the known differences between the receptive field sizes of nBP and BP neurons, provide evidence for the link between the spatial extent of electrosensory stimuli and short timescale correlation observed for superficial BP neurons.

### Feedback Inhibition Cancels Long Timescale Correlations

**Figure 7:**
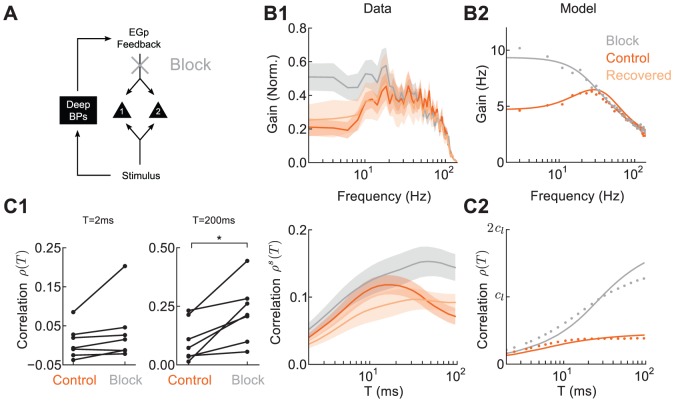
EGp feedback reduces correlations on long timescales when stimuli are global. **A** Schematic indicating block of feedback with CNQX in the ELL circuit. **B1**, Stimulus gain for individually recorded superficial BP neurons in control, block, and recovered conditions. Gain is normalized to output firing rate in the data. **B2**, Stimulus gain for model superficial neurons for global stimuli when feedback was intact or absent. **C1**, Left: Spike count correlation at 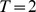 and 200 ms for paired recordings of superficial BP neurons. Right: Spike count correlation as a function of 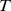 for individual recordings with a frozen stimulus in control, block, and recovered conditions. The standard error bars overlap for both the pre-drug and recovery curves, while they do not overlap with those for the block. Differences between control and recovered conditions could be due to incomplete drug washout or the preparation being in different states before and after the application of the drug. **C2**, Spike count correlation as a function of 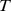 for model neuron pairs when feedback was intact or absent. Values are shown in units of input correlation in the local state 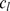.

We next tested how long timescale correlation is affected by recruitment of EGp feedback by global stimuli (Hypothesis 2). In our model, the EGp feedback was responsible for the decrease in low frequency stimulus gain and long timescale correlation in the global state. To experimentally confirm that this pathway was responsible for these effects, we pharmacologically blocked feedback from EGp to superficial neuron pairs (see Methods). We first tested whether attenuation of low frequency components of the stimulus gain was removed by the block. In experiments with global stimulation, we found that firing rates during the block were decreased significantly from the control condition (block: 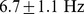; control: 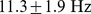, 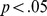, paired t-test). We remark that while the net impact of EGp feedback may be excitatory, the signal locked components of EGp feedback are thought to be inhibitory [46], consistent with our model. To correct for the change in firing rates across control and block conditions, we normalized the gain by firing rate to show the relative modulation of firing rate by the stimulus. The normalized gain increased at low frequencies, yet remained unchanged at high frequencies (Figure 7B1, compare orange and gray curves), consistent with model predictions (Figure 7B2). This effect was removed after a washout of the drug (Figure 7B1, compare orange and light orange curves).

The spike count correlations for simultaneously recorded superficial neurons in the global state with and without pharmacological block of feedback verified its role in shaping long timescale correlations. Specifically, the spike count correlations for 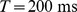 showed a significant increase during the block (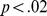, paired t-test), while correlations for 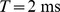 were similar (Figure 7C1; left). Due to the difficulty in obtaining paired recordings under pharmacological blockade, we further verified our theory with units recorded individually with frozen noise in the global state with and without pharmacological block of EGp feedback (Figure 7C1; right). Correlations at long timescales were increased during the block compared to control (Figure 7C1; left, compare orange and gray curves) and recovered to control values after drug washout (Figure 7C1; left, compare orange and light orange curves), consistent with our model (Figure 7C2). Thus, despite EGp feedback being a source of common synaptic input to a pair of superficial ELL pyramidal neurons, removing it during global stimulation increased the spike correlation between the neuron pair. In total, these data supported our second hypothesis: stimuli with large spatial extent recruit inhibitory feedback that cancels the input correlation expected from feedforward afferent projections.

### Correlation Shaping and Population Coding of Natural Electrosensory Scenes

We have presented a general mechanism for how spike train correlations from pairs of ELL pyramidal neurons are shaped by the spatial extent of an electrosensory signal. We explored the mechanism with simple noise signals categorized into either spatially local or global inputs. However, natural electrosensory scenes are complex, with a broad range of spatial and temporal scales. In this section, we speculate on how correlation shaping influences the population representation of natural electrosensory scenes.

Sensory systems must produce high fidelity representations of biologically relevant signals, while ensuring that distractor inputs do not contaminate the neural code. The ELL pyramidal neuron population is responsible for coding two distinct electrosensory inputs. First, electric fish routinely perform prey detection, tracking, and capture, during which prey organisms produce electric images with low frequency components (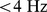) that stimulate a limited portion of the animal's electroreceptive field [55]. Second, electric fish use their electric organ to communicate with conspecifics, using signals that contain primarily high frequency components (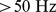) and drive a large region of the skin [56], [61]. However, these two signals often coexist with distractor inputs that the electrosensory system must ignore. Natural distractors arise from the superposition of background electric fields from many neighboring fish [62], or self generated signals from body and tail positioning [47]. These inputs consist of mostly low to mid range frequencies (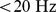) and drive a broad sensory area. A critical sensory computation in the ELL is the pyramidal neuron population faithfully locking to prey and communication signals, with minimal locking to distractor electrosensory inputs. The linear response analysis of the ELL-EGp network suggests that EGp feedback to the ELL plays an important role in this computation.

**Figure 8:**
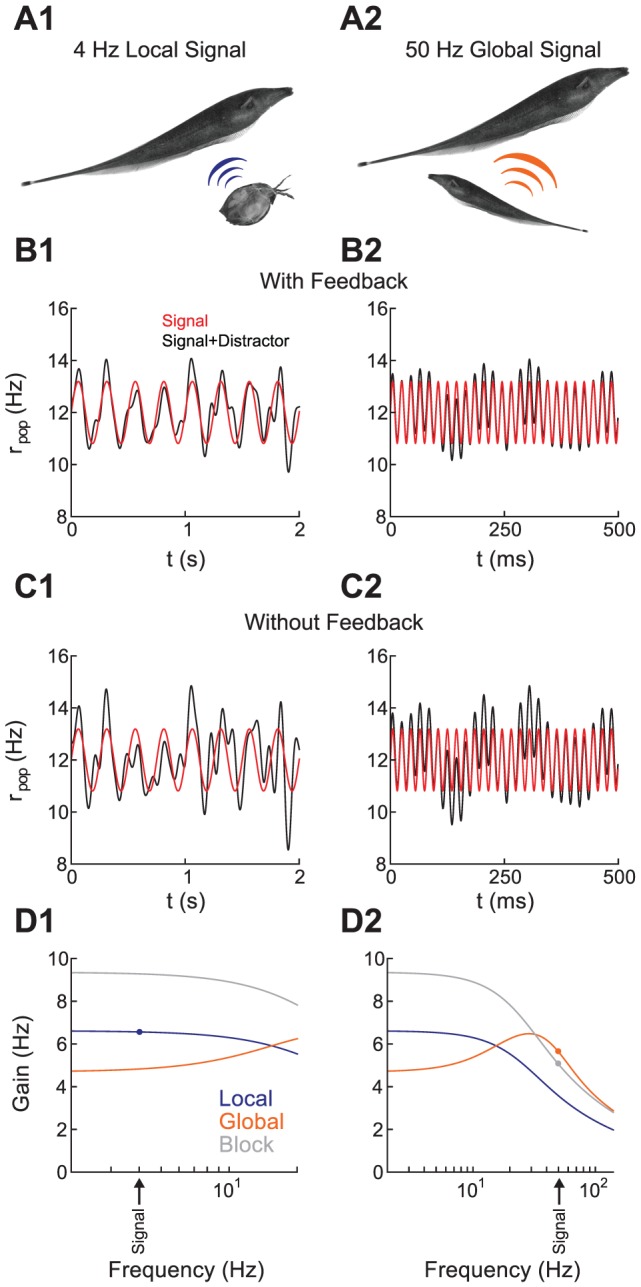
EGp feedback cancels the ELL population response to global distractor inputs but not prey or communication signals. **A1**, Schematic of response to a prey signal, which occupies a limited spatial extent and contains power at low frequencies. **A2**, Schematic of response to a communication call from a conspecific, which is a global, high frequency signal. **B1**, Average population firing rate for ELL neurons responding to a local, 4 Hz signal (red) and the same signal with 0–10 Hz distractor noise (black). The SNR was 2.3. **B2**, Same as B1, but with a global, 50 Hz signal. The SNR was 2.8. **C1**, Same as B1, but without EGp feedback. The SNR was reduced to 0.70. **C2**, Same as B2, but without EGp feedback. The SNR was reduced to 0.70. **D1**, ELL pyramidal neuron stimulus gain for local inputs (which do not recruit feedback) and global inputs with and without feedback. The frequency of the signal is marked. Note that because the distractor is a global 0–10 Hz signal, its transfer will be enhanced by the removal of feedback, reducing SNR (compare gray and orange curves). **D2**, Same as D1 but with a global, 50 Hz signal. Since the signal is high frequency, its stimulus gain is not substantially affected by feedback.

Using our linear theory, we calculated the response of a population of superficial BP neurons to mixed signal and distractor input, with and without EGp feedback. The signal was either a local 4 Hz sine wave (Figure 8A1–D1), or a 50 Hz global sine wave (Figure 8A2–D2). In both cases, the distractor input was 0–10 Hz broadband noise. The population response was modulated by the signal and the distractor, with relative strengths determined by the corresponding gain (Figure 8D). To test how EGp feedback affects the coding of relevant signals, we computed the signal to noise ratio (SNR) of this population response, defined as the ratio of the signal power integrated over all frequencies to the distractor power integrated over all frequencies. For both the 4 Hz local and 50 Hz global signals, the SNR was greater with feedback than without feedback (Figure 8B,C. SNR decreased from 2.3 to 0.70 for the 4 Hz local signal and from 2.8 to 0.70 for the 50 Hz global signal when feedback was removed). This is because EGp feedback was recruited by distractor input, attenuating any distractor induced correlation (low gain for distractor inputs in Figure 8D1,D2). In contrast, prey inputs lacked sufficient spatial power to recruit EGp feedback, meaning an EGp cancellation signal was not passed and ELL population stimulus gain was high (Figure 8D1). Communication calls have large spatial power, yet their high frequency power cannot be transmitted by the low pass parallel fiber pathway, again meaning ELL population stimulus gain was high (Figure 8D2). The ELL-EGp network was therefore capable of removing spurious correlations due to distractors while still coding for relevant signals.

## Discussion

Temporal shaping of correlated spiking activity has been observed in a variety of systems [7], [9], [19], [22]–[24]. We have shown that the spatial extent of an electrosensory signal controls the timescale of correlation between the spiking outputs of principal neurons in the ELL of weakly electric fish. Specifically, an increase in the spatial extent of a signal increased pairwise spike time synchronization, while simultaneously decorrelating long timescale rate co-variations. Using a combination of computational modeling and targeted physiological analysis, we identified that correlation shaping in the ELL is mediated both by an increase in the strength of feedforward afferent drive and the recruitment of a feedback pathway for spatially broad signals. Electric fish offer a neuroethologically inspired functional context for correlation shaping, where it promotes an accurate population representation of relevant signals, even in the presence of distractor inputs. The generic circuit features that support correlation shaping and its use in feature selective population temporal codes suggest that the basic principles exposed here may be at play in other neural systems.

### Correlation Shaping with Neural Architecture in the Electrosensory System

There has been extensive investigation of the gain shifts of single ELL pyramidal neurons between local and global stimulus configurations [26]–[30], [46]. These studies have shown that both feedforward and feedback mechanisms mediated these shifts. Indeed, pharmacological manipulations of descending feedback to the ELL provided strong evidence for its role in controlling gain shifts of single unit response at low frequencies [27], [29], [30], [46]. However, previous studies have shown that local stimuli only excited a fraction of the receptive field center [26], [29] and that spatial saturation of the receptive field center mediated the gain shifts of single unit response at high frequencies only by recruiting a greater fraction of feedforward afferent input [29], [30]. This importance of feedback activity prompted network models of the ELL and higher brain regions, and these models captured the sensitivity of single unit dynamics to the spatiotemporal structure of electrosensory stimuli [27], . However, the models relied on heretofore untested assumptions about the population spike train statistics of ELL pyramidal neurons. In parallel to these single-unit studies, other work presented simultaneous recordings from pairs of ELL pyramidal neurons showing significant stimulus evoked correlation in spike activity [63], and that the spike train correlation is sensitive to a stimulus' spatiotemporal structure [37]. However, these studies did not attempt to relate the dependence of pairwise statistics on stimulus structure to the extensive ELL single neuron experimental gain and network modeling literature. Our study merges the two avenues of research and shows that pairwise correlation shaping is related to gain shifts, as our linear response treatment of the ELL-EGp network model predicts. Thus, our analysis directly tests the proposed feedback mechanisms for single neuron response shifts [30].

Previous studies of the ELL have focused on the generation of oscillations due to feedback from area nP to pyramidal neurons (the direct feedback pathway) [27]. Theoretical studies have demonstrated that such oscillations arise from a combination of spatially correlated noise and delayed inhibitory feedback [28], [31]. Unlike neurons receiving closed-loop inhibitory feedback from nP, the superficial pyramidal neurons modeled in our study lack input from this direct pathway, and hence do not exhibit oscillations. Superficial neurons were excluded from the analysis in [27] and [28], so that the results of our study concern a cell class that is distinct from these previous studies. This distinction emphasizes the qualitative differences in the dynamics induced by open- and closed-loop feedback pathways.

We used well-characterized anatomical data and pharmacological manipulation to study the network architecture that codes for time-varying electrosensory stimuli. This is in contrast to techniques such as the generalized linear model [64] that statistically determine the spike response and network filters that generate a response to a sensory signal with fixed statistics. Our approach allowed us to study the response of the system in distinct stimulus conditions, with varying input statistics. Further, network coupling suggested clear architectural predictions for the mechanisms behind correlation shaping (hypotheses 1 and 2). These predictions were validated with a combination of the known heterogeneity of ELL feedfoward architecture (Figure 3), and a pharmacological blockade of feedback activity (Figure 7). Organisms exist in environments with ever-changing sensory statistics yet must code these environments, often with a single neural population. Our study shows how neural architecture can help shift the response dynamics of neural populations as signals change to better meet this computational need.

Our results also highlight how architectural differences may lead to differential population activity in different layers. Recently, it has been shown that synchronization between neurons in visual cortex is layer-dependent [65]. Furthermore, the cognitive demands of a task may control the recruitment of feedback and influence spike train correlations [66]. Our results demonstrate that both layer-specific recruitment of feedback and connectivity profiles influence correlated population activity.

Finally, theoretical communities have recently made some progress in understanding how network architecture combines with cellular dynamics to determine the correlation between pairs of cells [44], [67], [68]. However, the work is general, and a clear neural motivation to base a concrete example upon is lacking. Our study demonstrates that the electrosensory system offers a prototypical system where cellular dynamics, a clear feedforward/feedback architecture, and a single stimulus feature (spatial extent) interact to shape the temporal structure of pairwise spike train correlation.

### Decorrelating with Inhibition

The role of inhibition in neural circuits is a complex topic of study. Inhibition is linked to rhythmic, temporal locking between pairs of pyramidal neurons [32]. On fast timescales, inhibition is often thought to synchronize the activity of pairs of pyramidal neurons in both recurrent [10], [27], [33]–[35] and feedforward architectures [11], [32]. However, on longer timescales, inhibition mediates competitive dynamics between populations of pyramidal neurons, and as such may be a source of anti-correlated activity [24]. Recently, studies of densely coupled cortical networks with balanced excitation and inhibition [17], [18] and feedforward inhibitory cortical circuits [20], [69] have provided new insights into the role of inhibitory dynamics. In these studies, fluctuations in correlated excitation to a pair of pyramidal neurons are cancelled by correlated inhibitory dynamics, yielding a roughly asynchronous cortical state. This cancellation of correlation is similar to the one explored in our study responsible for the reduction of correlation for global stimuli. However, our study was motivated by a primarily feedforward sensory architecture in which an external signal can drive correlated activity.

The strengths of the electrosensory preparation allowed us to extend the correlation cancellation mechanism along two important directions. First, the ease in controlling the spatiotemporal properties of external stimuli allowed an analysis of the limitations of correlation cancellation. The diffuse ELL
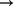
EGp feedforward path restricts correlation cancellation to signals with broad spatial scale, while the slow filtering by the parallel fiber pathway can only cancel correlations of low frequency stimuli. Second, the well segregated parallel fibers that mediate EGp feedback to the ELL permitted a pharmacological blockade of inhibition, directly providing evidence for correlation cancellation. The parallel fibers are a source of common input to pyramidal neurons, and a naive analysis would predict that their removal would thus decrease pyramidal neuron spike train correlation. Nevertheless, the blockade of parallel fiber inputs *increased* the spike train correlation, suggesting that the common inhibition interacts with the common feedforward afferent input in a destructive, rather than cooperative, manner.

Studies of neural codes often investigate the distinction between signal evoked, across-trial correlations and additional ‘noise’ induced, within-trial correlations [60]. Across-trial correlations are attributable to a dynamic locking of the spike train pairs to the common signal. Within-trial correlations measure the trial-to-trial co-variability of a pair of spike trains and may be increased relative to across-trial correlations due to common synaptic input to the neuron pair. These common fluctuations are often deleterious to cortical population codes [60], acting as a source of variability that cannot be removed through population averaging. The majority of our study presented simultaneously recorded spike train data which contains across-trial correlation as well as additional within-trial correlation. However, the shaping of correlation by the spatial profile of a stimulus was explained from knowledge of only of the across-trial correlation (Figures 1 and 2), and thus our ELL-EGp network model ignored other sources of correlation entirely. Our analysis did study the effects of irrelevant distractor inputs which can act as a source of noise, though originating from external signals rather than internal circuit mechanisms. We found that low frequency distractors that drive a substantial portion of the network recruit a cancellation signal. We therefore predict that within-trial correlations may be cancelled by a similar mechanism if they drive a large number of neurons synchronously. This may be the case when within-trial correlations are driven by the local field potential, which is often low frequency and widespread to populations of neurons [70], [71].

In summary, we have identified the combination of feedforward and feedback architecture that allows the spatial extent of a stimulus to shape the temporal correlations between the spike trains of pairs of electrosensory principal cells. Furthermore, correlation shaping allows populations of neurons to respond to stimuli that match a specific spatiotemporal profile and ignore distractor inputs. The generic architectural features of our network and the fact that sensory systems must filter irrelevant signals suggest that our findings may generalize to other systems.

## Supporting Information

Table S1 Model summary.
(PDF)

Text S1 Computation of cellular response function and power spectrum.
(PDF)
