# Supplementary material for: The Spatial Structure of Stimuli Shapes the Timescale of Correlations in Population Spiking Activity
Source: PLoS Comput Biol. 2012 Sep 13;8(9):e1002667. doi: 10.1371/journal.pcbi.1002667 (PMC3441501; doi:10.1371/journal.pcbi.1002667)
Supplement: Table S1 — Model summary. (PDF) [file pcbi.1002667.s001.pdf]

A. Model Summary	
**Populations**	Three: Deep (Dp), EGp, and Superficial (Sf)
**Topology**	Feedforward
**Connectivity**	All-to-all
**Neuron models**	Leaky integrate-and-fire (LIF)
**Channel model**	-
**Synapse model**	δ current-based and exponential current-based
**Plasticity**	-
**Input**	Gaussian white noise
**Measurements & Spike activity**	Spike times

B. Populations		
**Name**	**Elements**	**Size**
Dp	LIF	800
EGp	LIF	200
Sf	LIF	2

C. Connectivity			
**Name**	**Source**	**Target**	**Pattern**
Dp to EGp	Dp	EGp	All-to-all, weight $a_1 > 0$, δ-function
EGp to Sf	EGp	Sf	All-to-all, weight $a_2 < 0$, exponential

D. Neuron Model	
**Name**	LIF neuron
**Subthreshold dynamics**	$\frac{dV}{dt} = \frac{1}{\tau}(\mu - V) + I(t)$
**Spiking**	If $V(t) = V_{th}$, $V(t^+) = V_{re}$; t is marked as a spike

E. Input		
**Population**	**Recurrent Input Current**	**External input current**
Dp	-	$\sigma(\sqrt{c}\xi_s(t) + \sqrt{1-c}\xi_{i,n,Dp}(t))$
EGp	$a_1 \sum_{j=1}^{N_{Dp}} \sum_k \delta(t - t_{jk,Dp})$	$\sigma\xi_{i,n,EGp}(t)$
Sf	$a_2 \sum_{j=1}^{N_{EGp}} \sum_k \Theta(t - t_{jk,EGp}) e^{-(t-t_{jk,Dp})/\tau_s}$	$\sigma(\sqrt{c}\xi_s(t) + \sqrt{1-c}\xi_{i,n,Sf}(t))$

F. Parameters		
**Parameter**	**Description**	**Value**
N_{Dp}	Number of deep neurons	800
N_{EGp}	Number of EGp neurons	200
N_{Sf}	Number of superficial neurons	2
τ_{Dp}	Deep membrane time constant	10 ms
τ_{EGp}	EGp membrane time constant	10 ms
τ_{Sf}	Superficial membrane time constant	15 ms
μ_{Dp}	Deep bias	-56 mV
μ_{EGp}	EGp bias	-60 mV
μ_{Sf}	Superficial bias	-56 mV
V_{th}	Threshold voltage	-55 mV
V_{re}	Reset voltage	-65 mV
σ	Noise strength	1 mV
a_1	Deep to EGp synaptic strength	$7.6/N_{Dp}$ mV
a_2	EGp to Superficial synaptic strength	$-7.6/N_{EGp}$ mV
τ_s	EGp to Superficial synaptic time constant	5 ms
c_l	Local input correlation	0.1
c_g	Global input correlation	0.2
