# Supplementary material for: The Spatial Structure of Stimuli Shapes the Timescale of Correlations in Population Spiking Activity
Source: PLoS Comput Biol. 2012 Sep 13;8(9):e1002667. doi: 10.1371/journal.pcbi.1002667 (PMC3441501; doi:10.1371/journal.pcbi.1002667)
Supplement: Text S1 — Computation of cellular response function and power spectrum. (PDF) [file pcbi.1002667.s002.pdf]

Computing $A(f)$ and $Q_{ii}(f)$

In this section, we present the theoretical techniques that we use to compute the spike train cellular response function $A(f)$ and power spectrum $Q_{ii}(f)$ for the ELL model; these techniques are fully presented elsewhere [1] and we refer the reader there for further details.

Consider a leaky integrate and fire neuron model driven by a combination of a weak signal $s(t)$ and a white noise forcing $\xi(t)$:

$$\frac{dV}{dt} = \frac{\mu - V}{\tau} + \epsilon s(t) + \sigma\xi(t), \tag{1}$$

where μ is a drift term, σ is the intensity of a stochastic process, and $\epsilon << \sigma$. The voltage distribution $p(V,t)$ associated with the Eq. 1 obeys the Fokker-Planck equation [2]:

$$\frac{\partial p}{\partial t} = -\frac{\partial j}{\partial V} = \frac{\partial}{\partial V}\left[\left(\frac{V-\mu}{\tau} - \epsilon s(t)\right)p\right] + \frac{\sigma^2}{2}\frac{\partial^2 p}{\partial V^2}, \tag{2}$$

where $j(V,t)$ is the probability flux. The boundary conditions for the probability distribution and flux at threshold are $p(V_{th}) = 0$ and $j(V_{th},t) = r(t)$, where $r(t)$ is the firing rate. Furthermore, the flux obeys $j(V,t) = r(t)$ for $V \in [V_{re}, V_{th}]$ and 0 otherwise, when the system is stationary.

For $\epsilon = 0$ we obtain the steady state distribution $p_0(V)$ and flux $j_0(V)$ via:

$$\begin{aligned}
\frac{\partial p_0}{\partial V} &= -\frac{2}{\sigma^2}\left[j_0 + \frac{1}{\tau}(V-\mu)p_0\right], \\
\frac{\partial j_0}{\partial V} &= r_0\delta(V - V_{re}) - r_0\delta(V - V_{th}).
\end{aligned} \tag{3}$$

Numerically solving the above equations and using the normalization condition $\int_{-\infty}^{Vth} p_0(V)dV = 1$, we can solve for the steady state firing rate r_0.

To compute the second-order spike train statistics, we consider the time-dependent Fokker-Planck equation in the Fourier domain:

$$\begin{aligned}
\frac{\partial P}{\partial V} &= -\frac{2}{\sigma^2}\left[J + \frac{1}{\tau}(V-\mu)P\right], \\
\frac{\partial J}{\partial V} &= -2\pi i f P - R(f)\delta(V - V_{th}) + R(f)\delta(V - V_{re}),
\end{aligned} \tag{4}$$

where $P(V,f)$, $J(V,f)$, and $R(f)$ denote the Fourier transform of $p(V,t)$, $j(V,t)$, and $r(t)$, respectively. Further, $R(f)$ is computed with initial condition $V = V_{re}$. Solving this equation yields the Fourier transform of the first passage time density $D(f)$ [1]. For the stationary case, we compute the power spectrum using a well known relation from renewal theory relating passage time statistics to autocovariance [3]:

$$Q_{ii}(f) = r_0\left(1 + 2\Re\left[\frac{D(f)}{1 - D(f)}\right]\right), \tag{5}$$

where $\Re[\cdot]$ denotes the real component.

Finally, we compute the cellular response $A(f)$. We let $s(t) = e^{2\pi i f t}$ to provide a weak, periodic input to the neuron. Decomposing the probability density, flux, and firing rate into steady state and modulated components:

$$p = p_0 + p_1 e^{2\pi i f t},\ j = j_0 + j_1 e^{2\pi i f t},\ r = r_0 + r_1 e^{2\pi i f t}, \tag{6}$$

and then solving the Fokker-Planck equation in the Fourier domain for the time-dependent terms, we obtain a new set of equations:

$$\frac{\partial P_1}{\partial V} = -\frac{2}{\sigma^2}\left[J_1 + \frac{1}{\tau}(V-\mu) + \epsilon P_0\right],$$
$$\frac{\partial J_1}{\partial V} = -2\pi i f P_1 - R_1 \delta(V - V_{re}), \quad (7)$$

with boundary conditions $P_1(V_{th}, f) = 0$, and importantly the flux perturbation at spike threshold:

$$J_1(V_{th}, f) = A(f). \quad (8)$$

These equations were solved numerically [1] obtaining a solution for the cellular response $A(f)$.

The above theory provided the components $Q_{ii}(f)$ and $A(f)$ required to compute the spike count correlation coefficient between the pair of superficial ELL neuron outputs.

References

1. Richardson M (2008) Spike-train spectra and network response functions for non-linear integrate-and-fire neurons. Biol Cybern 99: 381–392.

2. Risken H (1996) The Fokker-Planck equation: Methods of solution and applications. New York: Springer, 488 pp.

3. Cox DR, Isham V (1980) Point processes. London: Chapman and Hall, 206 pp.
